# Transcriptional profile of *Paracoccidioides* spp. in response to itraconazole

**DOI:** 10.1186/1471-2164-15-254

**Published:** 2014-04-01

**Authors:** Benedito Rodrigues da Silva Neto, Patrícia Fernanda Zambuzzi Carvalho, Alexandre Melo Bailão, Wellington Santos Martins, Célia Maria de Almeida Soares, Maristela Pereira

**Affiliations:** 1 Departamento de Bioquímica e Biologia Molecular, Laboratório de Biologia Molecular, Instituto de Ciências Biológicas, ICBII, Campus II, Universidade Federal de Goiás, C.P. 131, 74001-970 Goiânia, GO, Brazil; 2 Instituto de Informática, Universidade Federal de Goiás, Goiânia, Goiás, Brazil

**Keywords:** *Paracoccidioides* spp., Transcriptional response, Itraconazole, Ergosterol

## Abstract

**Background:**

Itraconazole is currently used to treat paracoccidioidomycosis. The mechanism of action of azoles has been elucidated in some fungi, although little is known regarding its mechanism of action in *Paracoccidioides* spp. The present work focused on identification of regulated transcripts using representational difference analysis of *Paracoccidioides* spp. yeast cells treated with itraconazole for 1 and 2 h.

**Results:**

*Paracoccidioides Pb*01 genes up-regulated by itraconazole included genes involved in cellular transport, metabolism/energy, transcription, cell rescue, defense and virulence. *ERG*11, *ERG*6, *ERG*3, *ERG*5 and *ERG*25 were up-regulated at multiple time points. *In vivo* infection experiments in mice corroborated the *in vitro* results. Ergosterol levels and distribution were evaluated in *Paracoccidioides Pb*18 yeast cells, and the results demonstrate that both factors were changed in the fungus treated with itraconazole.

**Conclusion:**

To our knowledge, this is the first transcriptional analysis of *Paracoccidioides* spp. exposed to a triazole drug. Here acetyl seems to be intensively produced from different metabolic pathways to produce ergosterol by the action of ergosterol synthesis related enzymes, which were also affected in other fungi. Among the genes affected, we identified genes in common with other fungi, as well as genes unique to *Paracoccidioides Pb*01. Those genes could be considered target to new drugs. Voltage-gated Ca^2+^ alpha subunit (*CAV*), Tetracycline resistance protein (*TETA*) and Hemolisyn-iii channel protein (*HLYiii*) were found only here and a probably involvement with resistence to itraconazole could be investigated in the future. However our findings do not permit inference to current clinical practice.

## Background

*Paracoccidioides* spp., a complex of several phylogenetic species, is the agent of paracoccidioidomycosis (PCM). *Paracoccidioides* spp. is a thermodimorphic fungus, which grows in the soil as saprobic mycelium, resulting in the formation of propagules, which initiate infection in humans when inhaled into the respiratory tract. Subsequently, in the lung, the mycelia propagules develop into yeast cells [1]. PCM is endemic in Latin America [2], with 80% of cases reported in Brazil, where it is the eighth-leading cause of mortality among infectious and parasitic diseases, establishing it as a serious public health problem [3,4].

Itraconazole is suggested to be the best alternative for first-line therapy of PCM and should be administered over a long period [5]. Itraconazole is a triazole antifungal drug, which are multi-ringed synthetic compounds containing three nitrogen atoms in the azole ring. Mechanistically, the triazole drugs inhibit the synthesis of ergosterol, an essential component of fungal cell membranes, and cause abnormalities in the membrane permeability and consequently cell death [6]. Itraconazole and related azole derivatives act by blocking the ergosterol biosynthesis pathway through the inhibition of the fungal cytochrome P450 enzyme lanosterol demethylase (Erg11) [7].

The global response to azoles, including itraconazole, of fungi such as *Saccharomyces cerevisiae*[8], *Trichophyton rubrum*[9], *Aspergillus fumigatus*[10] and *Candida albicans*[11,12] has been studied using transcriptional and proteomic approaches. In general, the findings revealed both specific and nonspecific antifungal-induced changes in gene and protein regulation. There was an increase in expression of the genes involved in lipid, fatty acid and sterol metabolism, as well as genes involved in drug adaptation, including cell stress response, drug efflux and small molecule transport.

Despite of the importance of *Paracoccidioides* spp., nothing is known about the mechanism of itraconazole inhibition in this pathogen. Here, cDNA libraries were constructed to obtain expressed sequence tags (ESTs) of *Paracoccidioides* spp. The representational difference analysis (RDA) technique was used to identify changes in the transcriptional profile of *Paracoccidioides* spp. in response to itraconazole, with the aim of identifying the adaptative response of the fungus to the compound. Transcript levels were also measured during the infection process. In addition, the transcript levels of *ERG* genes, ergosterol levels and ergosterol localization were evaluated.

## Results

### Libraries characteristics

A total of 861 ESTs were successfully sequenced. From these, 224 up- and 208 down-regulated ESTs were obtained from yeast cells after incubation with itraconazole for 1 h, containing 55 singlets and 26 contigs for up-regulated transcripts and three singlets and 20 contigs for down-regulated ones. In addition, 230 up- and 199 down-regulated ESTs were obtained from yeast cells after incubation with itraconazole for 2 h, containing three singlets and 10 contigs for up-regulated and seven singlets and 12 contigs for down-regulated. The ESTs obtained were submitted to the National Center for Biotechnology Information (NCBI) database under accession numbers: LIBEST_028165 *Paracoccidioides Pb*01 itraconazole 1 h up Library, LIBEST_028164 *Paracoccidioides Pb*01 itraconazole 1 h down Library, LIBEST_028167 *Paracoccidioides Pb*01 itraconazole 2 h up Library and LIBEST_028166 *Paracoccidioides Pb*01 itraconazole 2 h down Library.

The ESTs were processed using the Blast2GO program, which allowed us to annotate and identify the different functional groups. The functional classification was based on the homology of each EST, considering e-values ≤10^-5^ significant, using BLASTx against the GenBank non-redundant database and the MIPS functional annotation scheme (Table 1). The analyses indicated the presence of transcripts from different functional categories: metabolism/energy, transcription, cell rescue, defense and virulence, protein synthesis and biogenesis, protein fate, cellular transport, biogenesis of cellular components and cellular communication.

**Table 1:** **Genes differentially expressed in *Paracoccidioides *** **in response to treatment with itraconazole**

***Functional category***	***Gene product***	***Best hit/Paracoccidioides genome locus***	***e-value***	***Number of occurrences***^***a***^	
				**1 h**	**2 h**
**Metabolism/Energy**	3-deoxy-7-phosphoheptulonate synthase (*DAHP*)	PAAG_03237	9.2e-29	+2	
	Cysteine desulfurase (*CYSD*)	PAAG_05850	2.2e-58	+22	
	Betaine aldehyde dehydrogenase (*BADH*)	PAAG_05392	2.1e-19	+2	
	NADP-specific glutamate dehydrogenase (*GDH*)	PAAG_07689	1.6e-26	+1	
	NAD dependent epimerase dehydratase (*EDH*)	PAAG_05580	1.6e-30	+1	
	Succinyl 3-ketoacid-coenzime A transferase (*SCOT*)	PAAG_05093	2.6e-17	+1	
	Ribulose-phosphate 3-epimerase (*RP3E*)	PAAG_01632	6.5e-42	-17	
	Aconitase (*ACO*)	PAAG_05328	5.8e-18	-2	
	D-amino-acid oxidase (*DAAO*)	PAAG_02361	2.4e-38	-3	
	Er-associated proteolytic system protein (*ERAD*)	PAAG_04633	8.6e-40	-4	
	Isovaleryl-CoA dehydrogenase (*IVD*)	PAAG_06830	1.0e-23	+4	
	Acyl-CoA dehydrogenase (*ADH*)	PAAG_05211	1.0e-30	+1	
	Acyl-CoA dehydrogenase (*ADH*)	PAAG_01222	2.1e-19	-8	
	Hormone-sensitive lipase (*LIPE*)	PAAG_06218	7.6e-37	+1	
	Pyruvate kinase (*PK*)	PAAG_06380	3.1e-31	+1	
	Aldehyde dehydrogenase (*ALDH*)	PAAG_05249	1.0e-44	-4	
	Glutamine amidotranferase subunit pdxT (*GLAT*)	PAAG_07505	1.3e-23	-5	
	ATP synthase f0 subunit 9 (*ATPS9*)	PAAG_12009	3.0e-17		+22
	Short chain dehydrogenase (*DHS-14*)	PAAG_04787	3.2e-48	+1	
**Transcription**	Transcription factor (*STEA*)	PAAG_00406	8.4e-50	+3	
	Isoform cra_b	PAAG_05467	3.2e-15	+1	
	Fator transcrição tipo CCCH	PAAG_02735	4.2e-27	+1	
	Pirin (*PIR*)	PAAG_04726	2.5e-52	+7	+47
	RING finger protein (*RNF*)	PAAG_06129	1.5e-18	+2	
	Apses transcription (*APSES*)	PAAG_02379	1.6e-30	+1	
	mRNA-nucleus export ATPase	PAAG_04548	4.0e-45	-27	
	C6 transcription factor (*CTFIB*)	PAAG_01359	4.5e-65	-12	
**Cell rescue, Defense and Virulence**					
	Survival factor1 (*SVF1*)	PAAG_02425	4.7e-36	+1	
	Gluthatione S-transferase (*GST*)	PAAG_03931	1.0e-33	+1	
	Vanadate resistence protein	PAAG_03940	9.4e-54	+2	
	Heat shock protein (*STI1*)	PAAG_06811	2.2e-24	+2	
	Heat shock protein *(HSP10*)	PAAG_05142	6.5e-32	+1	+2
	Heat shock protein (*HSP30*)	PAAG_00871	5.4e-52	-26	-33
	Heat shock protein (*HSP70*)	PAAG_08003	4.4e-40		+3
	Heat shock protein (*HSP60*)	PAAG_08059	4.3e-55		-12
**Protein synthesis and biogenesis**					
	ATP-dependent RNA helicase (*ELF4A*)	PAAG_00689	1.3e-24	+7	
	Serine threonine-protein kinase (*SRK1*)	PAAG_06726	7.6e-66	-2	
	40S ribosomal protein S4 (*RPS4*)	PAAG_03816	7.3e-37		+2
**Protein fate (folding, modification, destination)**					
	Ubiquitin-protein ligase (*UBI*)	PAAG_02632	3.7e-11	+2	
	WD repeat containing protein (*WDR*)	PAAG_00103	1.0e-25	+1	
	Ubiquitin thioesterase (*OTU1*)	PAAG_08841	1.0e-32	-5	
	Ubiquitin fusion degradation protein (*UFD*)	PAAG_01475	1.0e-62		+1
	Proteasome component (*PREP6*)	PAAG_07802	6.1e-5	+1	
**Cellular transport, transport facilities and transport routes**					
	Tetracycline resistance protein (*TETA*)	PAAG_01353	1.0e-56	+13	
	Mfs transporter (*MFS*)	PAAG_02191	7.3e-56	+9	
	Nucleoporin (*SONB*)	PAAG_02655	1.1e-36	+1	
	Voltage-gated Ca^2+^ alpha subunit (*CAV*)	PAAG_01353	9.9e-15	+1	
	Sodium-dependent phosphate transporter (*SPIT*)	PAAG_03892	1.5e-11	+1	
	Zinc finger membrane protein (*DHHC*)	PAAG_06616	6.6e-53	-4	
	autophagy regulatory protein	PAAG_04970	2.6e-33	-2	
	*GPR1/FUN34/YAAH* family protein	PAAG_08587	1.2e-47	-13	-5
	Carnitine/acyl carnitine carrier (*CAR*)	PAAG_03452	1.7e-30	-18	-89
	General secretion pathway protein.	PAAG_05009	2.7e-60	-10	
	Family integral membrane protein (*IMP*)	PAAG_03183	4.1e-50		+1
	Hemolisyn-iii channel protein (*HLYiii*)	PAAG_01871	6.0e-34		+3
	Integral membrane *MPV17*/*PMP*22	PAAG_02868	6.6e-37		-2
	Vesicular fusion protein (*SEC17*)	PAAG_06233	2.8e-77		-1
**Biogenesis of Cellular Components (cell wall/membrane)**					
	Chitin synthase regulator 2 (*CHSr*)	PAAG_04860	2.0e-17	+3	
	Oxysterol-binding protein (*OSBP*)	PAAG_06807	3.6e-45		+6
	Diacylglycerol *o*-acyltransferase (*DGAT*)	PAAG_07527	1.4e-64	+1	
	Phosphatidyl synthase (*PHS*)	PAAG_03571	6.5e-34	+1	
**Cellular communication/Signal transduction mechanism**					
	FluG Domain-containing protein	PAAG_05486	1.0e-53	+1	
	Leucine –rich repeat Igi member 4 (*LGI4*)	PAAG_00833	1.0e-25	+1	
	Conserved Lysine protein (*LYS*)	PAAG_03092	6.2e-43	+2	
**Unclassified protein**	Conserved hypothetical protein	PAAG_02735	1.3e-39	+2	
	Conserved hypothetical protein	PAAG_01353	1.3e-18	+6	
	Conserved hypothetical protein	PAAG_07364	5.8e-30	+4	
	Conserved hypothetical protein	PAAG_00520	3.9e-27	+3	
	Conserved hypothetical protein	PAAG_02379	1.5e-19	+2	
	Conserved hypothetical protein	PAAG_02210	1.8e-37	+6	
	Conserved hypothetical protein	PAAG_02236	1.0e-15	+1	
	Conserved hypothetical protein	PAAG_03559	4.8e-47	+1	
	Conserved hypothetical protein	PAAG_03596	4.4e-83	+3	
	Conserved hypothetical protein	PAAG_08759	1.5e-63	+2	
	Conserved hypothetical protein	PAAG_07907	1.0e-42	+2	
	Conserved hypothetical protein	PAAG_04000	1.0e-44	-4	
	Conserved hypothetical protein	PAAG_06816	1.7e-8		-2
	Conserved hypothetical protein	PABG_06807	1.0e-51		-6
	Conserved hypothetical protein	PAAG_01871	1.0e-51		-2
	Conserved hypothetical protein	PAAG_07034	3.4e-33	+2	
	Conserved hypothetical protein	PADG_04444	6.7e-17		+2
	Hypothetical protein	PAAG_02259	1.0e-18	-3	
	Hypothetical protein	PAAG_02991	4.9e-37	+1	
	Domain-containing protein (*DUF1688*)	PAAG_04000	7.5e-17	+2	

^*a*^Occurrences are expressed as the fold change relative to the value for the nontreated control; +, induction; -, repression.

### Global gene expression monitoring in *Paracoccidioides Pb*01 upon itraconazole treatment

**Figure 1:**
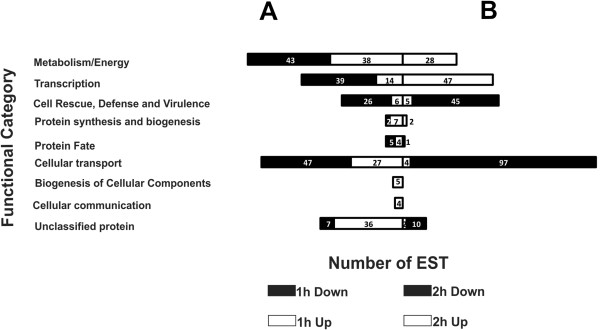
**Functional classification of genes responding to itraconazole in *Paracoccidioides. ***cDNAs obtained from RNAs from yeast cells after incubation with itraconazole for 1 h **(A)** and 2 h **(B)**. The numbers of ESTs are indicated with white bar segments for the up-regulated genes and black bar segments for the down-regulated genes. The annotation of genes was performed using the Blast2GO program with a cut-off for significant homology of ≤ 1e^-5^. Sequences were grouped into functional categories according to their classification in the MIPS functional catalog. Additionally, sequences were grouped into functional categories using the PEDANT 3 database. Each functional class is represented as a segment and expressed as a number of ESTs in each library.

A total of 86 genes were differentially expressed upon exposure to itraconazole, of which 55 were up-regulated and 31 were down-regulated. ESTs obtained from 1 h treatment with itraconazole were clustered into functional classes which were defined as metabolism/energy (26.12%); transcription (17.09%); cell rescue, defense and virulence (10.32%); protein synthesis and biogenesis (2.90%); protein fate (2.90%); cellular transport (23.87%); biogenesis of cellular components (1.61%); cellular communication (1.29%); and unclassified proteins (13.87%). ESTs from 2 h samples were clustered into functional classes which were defined as: metabolism/energy (11.61%); transcription (19.5%); cell rescue, defense and virulence (20.74%); protein synthesis and biogenesis (0.82%); protein fate (0.41%); cellular transport (41.90%); and unclassified protein (4.97%) (Figure 1).

It were found genes precursors of acety groups, from different metabolic pathways, such as acyl-CoA dehydrogenase (*ADH*), isovaleryl-CoA dehydrogenase (*IVD*), pyruvate kinase (*PK*) and cysteine desulfurase (*CYSD*).

In addition, genes precursors to the components of membrane and cell walls were found, such as phospholipids and carbohydrates, as well as genes related to detoxification. These components are diacylglycerol *o*-acyltransferase (*DGAT*), chitin synthase regulator 2 (*CHSr*), hemolysin-iii channel protein (*HLYiii*), tetracycline resistance protein (*TETA*), voltage-gated Ca^2+^ alpha subunit (*CAV*) and the MFS transporter.

### Expression profiles of genes in *Paracoccidioides Pb*01 yeast cells

**Figure 2:**
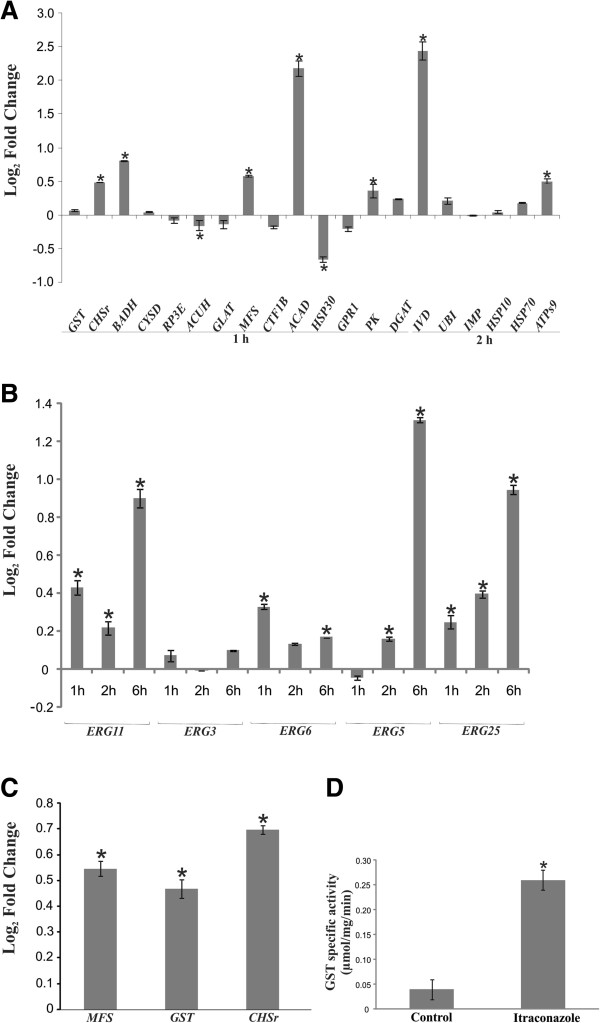
**Relative fold change for genes determined by qRT-PCR.** The gene expression profile for **(A)** twenty genes listed in Table 2, **(B)** the ergosterol pathway genes and **(C)***in vivo* samples of *Paracoccidioides* recovered directly from systemically infected mouse spleens. Changes in gene expression levels were calculated by relative standard curve method using the control, untreated samples as the calibrator. **(D)** GST activity was measured in protein extract from *Paracoccidioides Pb*18 yeast cells grown in the presence or absence of itraconazole. Each error bar represents the standard error of the mean (±SE) from three independent experiments performed in triplicate, and significant fold-changes are denoted by asterisks in the figure (*p ≤ 0.05). Data were normalized to the transcript encoding the α-tubulin protein. Student’s *t* test was used for statistical comparisons.

Confirmation of the expression levels of the ESTs found in the redundancy analysis was performed by qRT-PCR analysis, including *Paracoccidioides Pb*01 glutathione S-transferase (*GST*), (*CHSr*), betaine aldehyde dehydrogenase (*BADH*), *CYSD*, ribulose-phosphate 3-epimerase (*RP3E*), carnitine/acyl-carnitine carrier (*CAR*), C6 transcription factor (*CTFIB*), *ADH*, heat shock protein (*HSP30*), *GPR1/FUN34/YAAH* family protein, *PK*, *DGAT*, *IVD*, ubiquitin-protein ligase (*UBI*), family integral membrane protein (*IMP*), *HSP10*, *HSP70* and ATP synthase f0 subunit 9 (*ATPS9*). These genes were chosen because of their high frequency or as representatives of different functional categories. Differential expression profiles of genes corroborated RDA data (Figure 2A).

### Analysis of ERG transcripts by qRT-PCR

Because *ERG* transcripts and proteins levels were changed in the presence of azoles in fungi such as *T. rubrum*[9], *S. cerevisiae*[8], *C. albicans*[11,12] and *A. fumigatus*[13] we investigated whether ergosterol synthesis-related transcripts such as lanosterol 14 α-demethylase (*ERG11*), C5,6-desaturase (*ERG3*), delta-24-sterol C-methyltransferase (*ERG6*), C-22 sterol desaturase (*ERG5*) and C-4 methyl sterol oxidase (*ERG25*) were changed in *Paracoccidioides Pb*01 after 1 h, 2 h and 6 h of exposure to itraconazole using specific oligonucleotides in qRT-PCR. The results showed that all transcripts were increased at all time points (Figure 2B).

### *Paracoccidioides Pb*18 transcripts identified in mice treated with itraconazole

We investigated whether the regulated transcripts identified by RDA experiments using *Pb*01 also occurred in another cryptic species, *Pb*18*, in vivo.* Balb/c mice infected with *Paracoccidioides Pb*18 were treated with itraconazole, and spleens were removed. The treatment with itraconazole reduced the fungal burden 42% in the spleens. RNAs extracted from recovered fungus were analyzed in qRT-PCR experiments using *MFS*, *GST* and *CHSr* genes. In agreement with the RDA data, all the evaluated genes were up-regulated in spleen fungal samples after treatment with itraconazole (Figure 2C).

### GST-specific activity correlates with transcriptional data

Because *GST* transcripts were up-regulated in our study and are described in the literature as important for the detoxification of many different xenobiotics [14], we evaluated the GST-specific activity in protein extracts of fungus grown in the presence of itraconazole. GST-specific activity in the presence of itraconazole (0.26 μmol/mg/min) was 6.5 times higher than in the absence of itraconazole (0.04 μmol/mg/min) (Figure 2D).

### Analysis of the ergosterol level

Because transcript levels of ergosterol pathway components were changed in the presence of itraconazole, we evaluated if itraconazole could disturb the total intracellular level of ergosterol. The method for quantification of ergosterol used here takes advantage of the unique four-peak spectral absorption pattern produced by extracted sterols between 240 and 300 nm. Comparing the scans obtained from control (1.0 g of ergosterol/g yeast cells to *Pb*01; 1.2 g of ergosterol/g yeast cells to *Pb*18) and the corresponding itraconazole-exposed cultures (0.80 g of ergosterol/g yeast cells to *Pb*01; 0.62 g of ergosterol/g yeast cells to *Pb*18), a decrease of 39% and 48.6% was identified in the ergosterol content of *Paracoccidioides Pb*01 and *Pb*18 yeast cells, respectively grown in the presence of itraconazole.

### Effect of itraconazole on ergosterol localization in *Paracoccidioides Pb*01 and *Pb*18 yeast cells

Because itraconazole induces changes in transcript levels in the ergosterol pathway and disturbs the total intracellular ergosterol content [15], the localization of ergosterol molecules was assessed in *Paracoccidioides Pb*01 and *Pb*18 yeast cells. Ergosterol was detected by its ability to bind to the dye filipin. This characteristic has been used to detect ergosterol in dimorphic fungi [16], yeasts, filamentous fungi [17,18] and mammalian cells [19].

**Figure 3:**
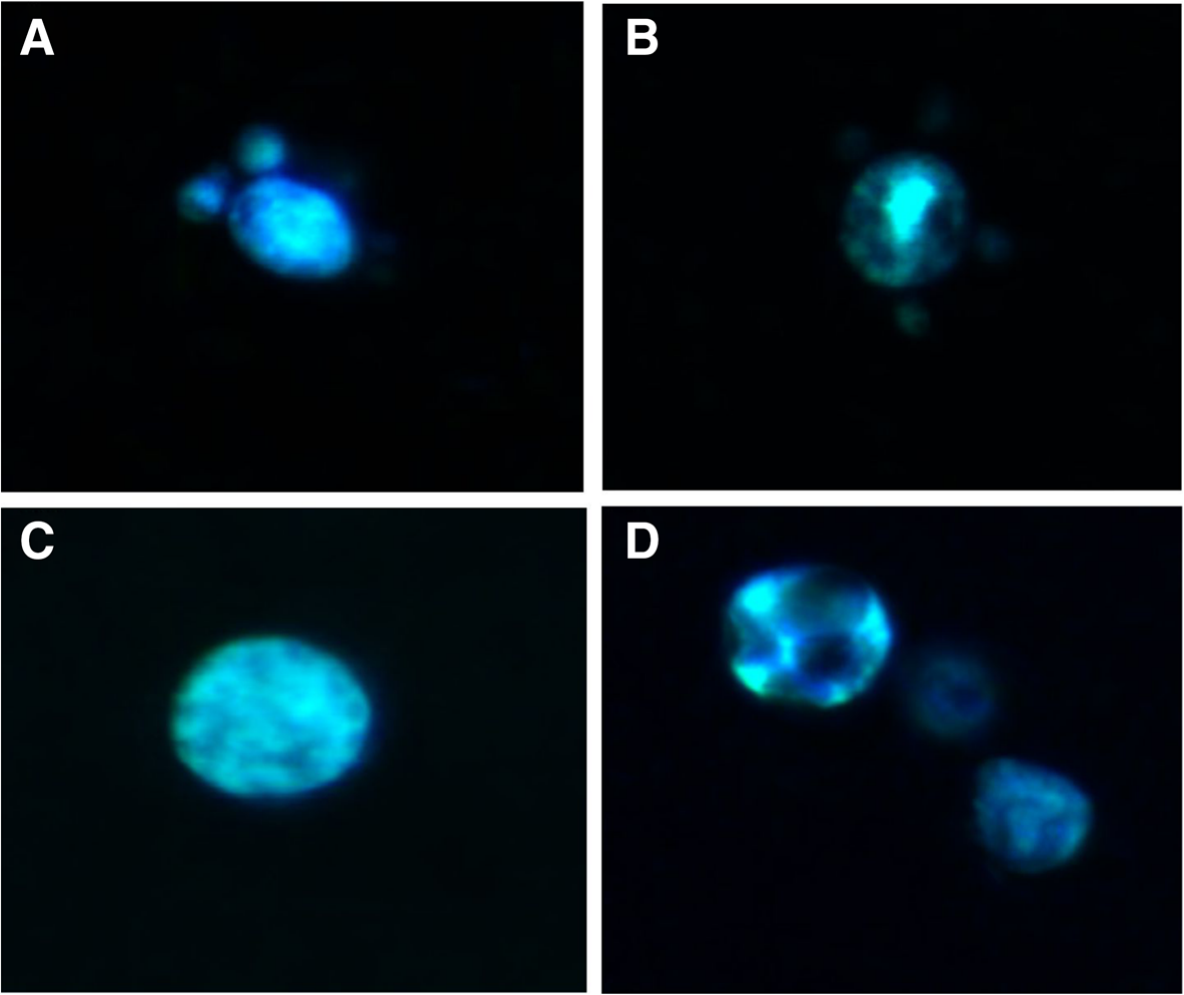
**Sterol distribution in *Paracoccidioides *spp..** Yeast cells were fixed, stained with filipin and observed by fluorescence microscopy. Staining in the control *Pb*01 **(A)** and *Pb*18 **(C)** cells was diffuse with homogeneous labeling. *Pb*01 **(B)** and *Pb*18 **(D)** cells treated with itraconazole displayed heterogeneous fluorescence.

The distribution of ergosterol on the surface of *Paracoccidioides Pb*01 and *Pb*18 yeast cells treated with itraconazole was strikingly different from that observed in the control untreated cells. Control cells showed a homogeneous fluorescence distribution (Figure 3A and C). In contrast, the cells treated with itraconazole displayed dark regions without filipin fluorescence (Figure 3B and D).

### A model for the *Paracoccidioides* spp. adaptation to the itraconazole

**Figure 4:**
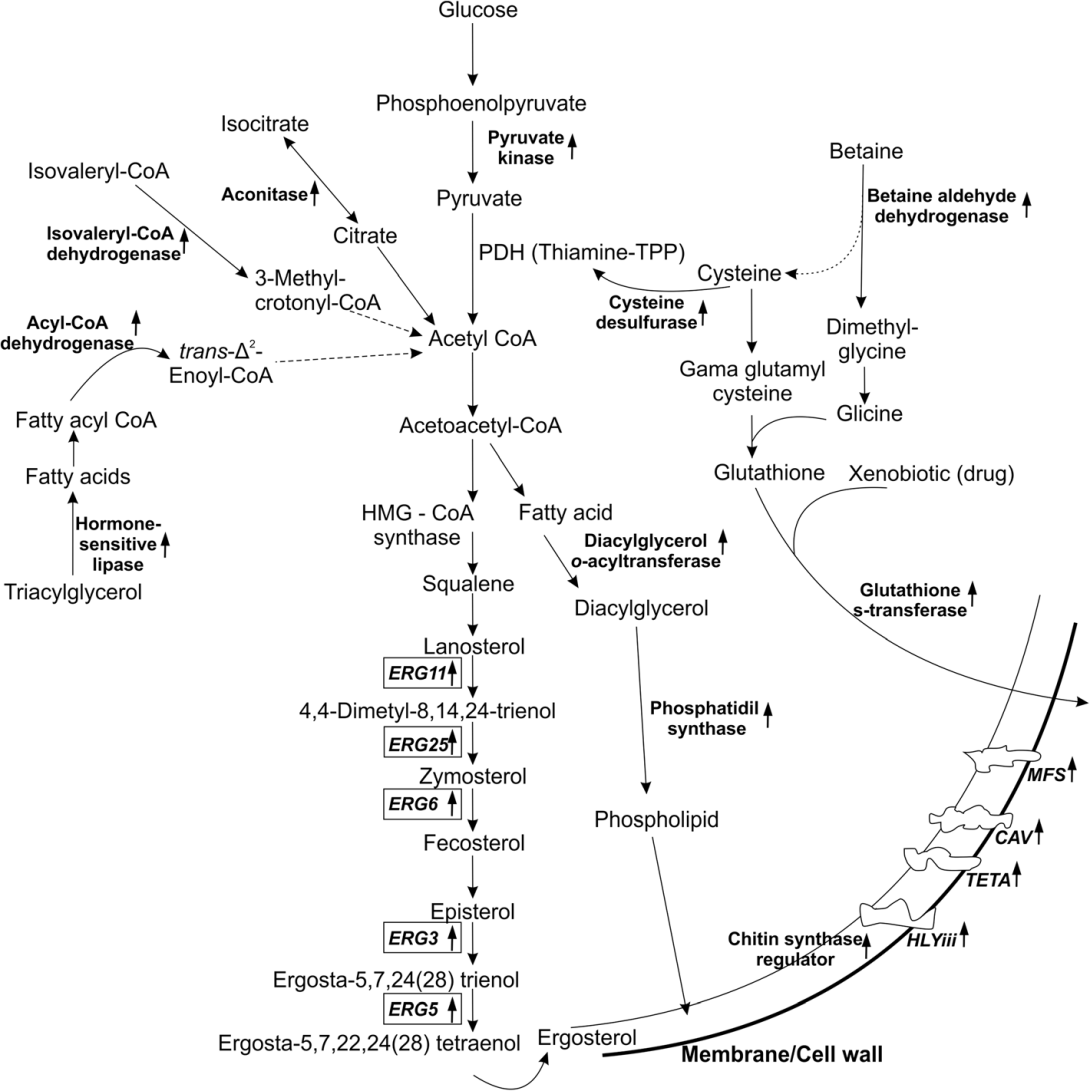
**Hypothetical model for the mode of action of itraconazole against *Paracoccidioides*.** The up-regulation of transcripts such as hormone-sensitive lipase, *ADH*, *IVD* and *ACO* from different metabolic pathways would produce acetyl CoA that would be used for ergosterol synthesis by *ERG* enzymes. Acetyl CoA would produce phospholipids for the membrane by the action of *DGAT* and *PHS*. The induction of *BADH* and *CYSD* would lead to production of thiamine, a cofactor of *PDH*, which would also produce acetyl CoA. *GST* would conjugate glutathione to xenobiotics and would remove itraconazole from the cell using transporters, allowing for detoxification.

The most prominent adaptations undergone by *Paracoccidioides* spp. during exposure to itraconazole are summarized in Figure 4. See the Discussion for details.

## Discussion

**Figure 5:**
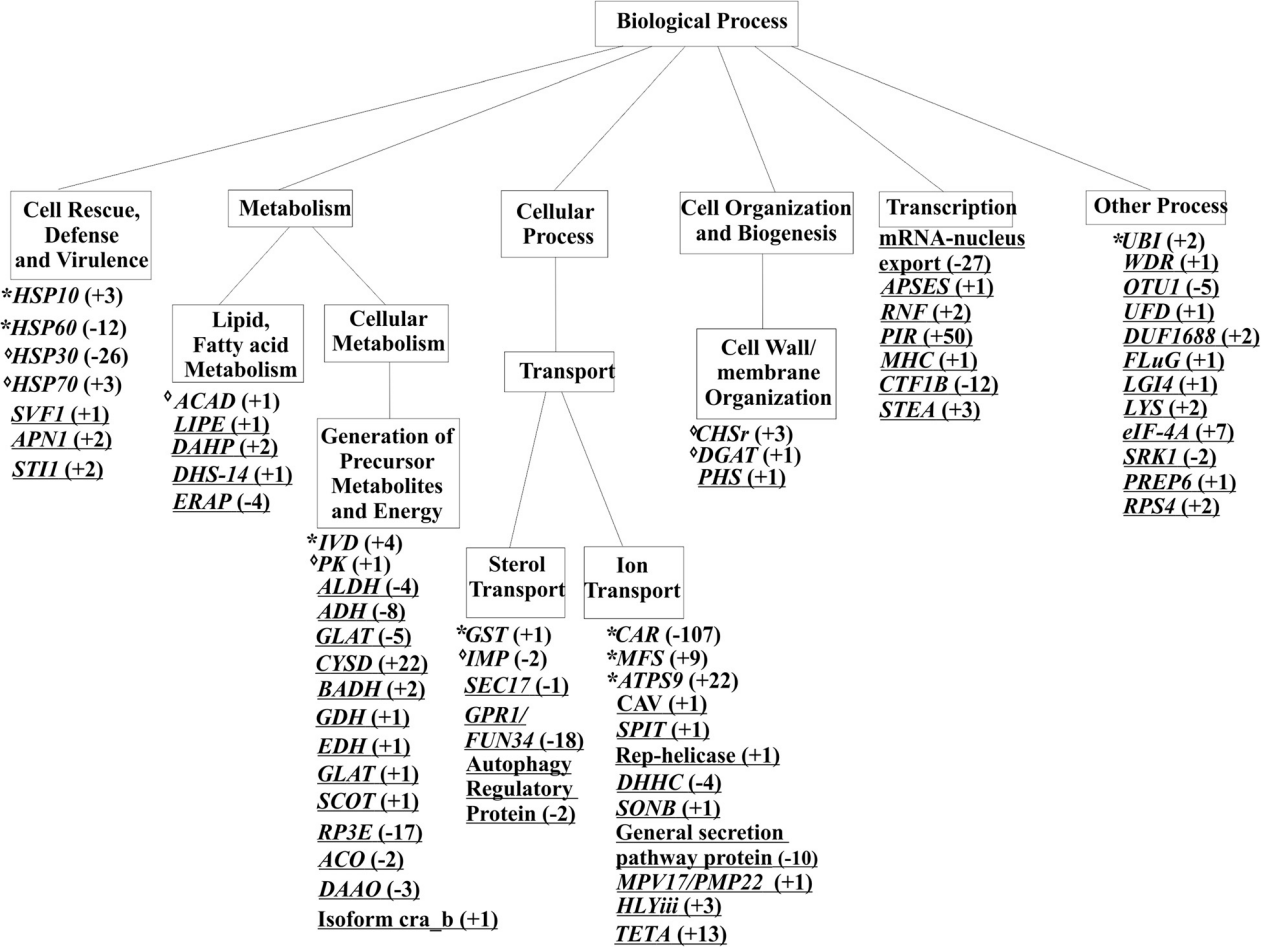
**Distribution of genes responding to itraconazole in *P. brasiliensis *isolate *Pb *01.** Data are shown for a subset of genes that were significantly up or down regulated (e-values ≤10^-10^). The search for functional categories was performed by using the Blast2GO program that joints in one application GO annotation based on similarity searches with statistical analysis and highlight visualization on directed acyclic graphs. GO terms shown are those that were considered significantly over represented by the analysis. Sequences were grouped in functional categories according to the classification of the MIPS functional catalog (Munich Center for Protein Sequences; http://mips.gsf.de/). Specific genes for *P. brasiliensis* isolate *Pb*01 are underlined, genes found in other fungi when exposed to itraconazole and other azoles are represented with * and represented with ◊, respectively. Numbers in parentheses represent changes in gene expression. Positive signal indicate induction, and negative indicate repression.

Among the *Paracoccidioides Pb*01 genes regulated by itraconazole were those involved in cellular transport, metabolism/energy, transcription, cell rescue, defense and virulence. Similar and different groups were also observed in other fungi in response to different azoles [8,9,11,12,15] (Figure 5). Among the genes affected, we identified genes in common with other fungi, as well as genes unique to *Paracoccidioides*. In fact few *Paracoccidioides* spp. genes were shared with genes from other fungi. This could be due to different techniques and classes of azoles used in the works. The comparison with ither fungi show that cell processes related to stress response, xenobiotic efflux are trigered upon itraconazol in different fungi. The genes exclusively regulated in *Paracoccidioides* spp. reveal that fungi response to drugs can partially involve specific processes that may be related to different sensibility of differetn fungi to itraconazol treatment. This could be due to different techniques and classes of azoles used in the works.

Although *ERG* genes were not identified in the RDA experiments, qRT-PCR results showed that *ERG11*, *ERG3*, *ERG6*, *ERG5* and *ERG25* genes were temporally regulated, particularly after longer contact with the drug (6 h). Acetyl is a carbon donator in the cell production of ergosterol [8]. Acetyl CoA seems to be intensively produced due to up-regulation of transcripts from different metabolic pathways, including lipid degradation by hormone-sensitive lipase (*LIPE*) and *ACAD* and amino acid metabolism by *IVD*. Acetyl CoA pool increasing in the cell is optimized with reduction of aconitase trasncripts (*ACO*), once this enzymes participates of the acetyl-CoA oxidation in TCA. In addition, the induction of *BADH* and *CYSD* could lead to production of thiamine, a cofactor to pyruvate dehydrogenase (*PDH*), which produces acetyl CoA from pyruvate, whose production is increased by the action of pyruvate kinase (Figure 4).

Ergosterol is produced by the action of erg enzymes [20]. Here, the action of itraconazole on ergosterol biosynthesis and its distribution on *Paracoccidioides Pb*01 and *Pb*18 yeast cells surface was documented. Ergosterol is an essential component of fungal plasma membranes; it affects membrane permeability and the activities of membrane-bound enzymes. This sterol is a major component of secretory vesicles and has an important role in mitochondrial respiration and oxidative phosphorylation [21,22]. It can thus be expected that changes in ergosterol levels and in sterol structure could influence the activities of several metabolic pathways. The mechanism responsible for the global up-regulation of *ERG* genes in response to azoles remains unclear. One theory postulates that depletion of ergosterol or another sterol formed late in the pathway increases global *ERG* expression; another argues that accumulation of an early substrate or toxic sterol by product induces *ERG* expression [23].

The correlation between cell wall integrity and perturbation of the ergosterol pathway in *T. rubrun* suggests that changes in the cell wall may compensate for stress in the plasma membrane [9]. The phospholipid level in the cell membrane seems to be affected in *Paracoccidioides Pb*01, as indicated by up-regulation of *DGAT* and phosphatidyl synthase (*PHS*), which produce phospholipids. *DGAT* has been found in several transcriptomes to date, indicating it may be important for the fungi response to azoles [15,24].

*CHS* and their regulatory genes are important for the growth and virulence of human fungal pathogens, including *C. albicans*[25,26]. It has been observed that high ergosterol levels can inhibit chitin synthases, whereas *C. albicans* mutants with low ergosterol content showed increased levels of chitin synthesis [27]. *CHSr* was up-regulated in *Paracoccidioides Pb*01 in the presence of itraconazole.

Glutathione S-transferases, which are important for the detoxification of many xenobiotic compounds, are a family of multifunctional enzymes that play a role in cellular detoxification and excretion of a wide variety of xenobiotic substances [14]. It has been reported that Glutathione S-transferases correlate with fungi defense in response to damage caused by oxidative stress, xenobiotics and antifungal compounds [28]. *GST* was up-regulated in *Paracoccidioides* in the presence of itraconazole.

In *Paracoccidioides Pb*01, genes encoding several classes of transporters were up-regulated upon exposure to itraconazole. MFS transporter and *TETA*, for example, have been implicated in azole resistance [29]. Drug resistance is often associated with the overexpression of genes encoding efflux pumps, which is presumed to prevent intracellular accumulation of itraconazole in fungus [9,30]. The up-regulation of *Paracoccidioides Pb*01 *MFS*, *GST* and *CHSr* transcripts also occur *in vivo*, as demonstrated here by qRT-PCR using RNAs extracted from spleens of mice.

It should be noted that a number of genes involved in small molecule transport, especially in ion transport, were differentially expressed in *Paracoccidioides Pb*01 in response to itraconazole. Up-regulated genes included *CAV*, *IMP* and *HLYiii*. Down-regulated genes included (*CAR*) and integral membrane *MPV17/PMP22*. The inhibition of ergosterol, which is an essential component of fungal biological membranes, including the plasma membrane, can lead to destabilization of the membrane, leakage of cellular components and influx of extracellular ingredients. Therefore, the regulation of transporter genes is necessary to maintain ionic homeostasis within the fungal cell when membranes are damaged by itraconazole [9,31].

## Conclusion

This is the first study to analyze the changes in the *Paracoccidioides* spp. gene expression profile following triazole exposure. Among the genes affected, we identified genes unique to *Paracoccidioides Pb*01, as well as genes in common with other fungi. *In vitro* results were validated by *in vivo* experiments. The results obtained here should assist in understanding the mode of action of itraconazole in *Paracoccidioides* spp*.*

## Methods

### Culture and cell viability

*Paracoccidioides Pb*01 and *Pb*18 have been studied at our laboratory previously [32,33]. *Pb*01 and *Pb*18 yeast phase was maintained *in vitro* by subculturing at 36°C in Fava Netto’s semisolid medium [34] every seven days. Fava Netto’s semisolid medium components were as follows: 1% (w/v) peptone, 0.5% (w/v) yeast extract, 0.3% (w/v) proteose peptone, 0.5% (w/v) beef extract, 0.5% (w/v) NaCl, 4% (w/v) glucose and 1.2% (w/v) agar, pH 7.2. The determination of IC_50_ was performed according to Santos et al. [23] and in accordance with the macro dilution method described in the Clinical and Laboratory Standards Institute (CLSI) M27-A2(2005), with modifications. To determine the IC_50_, yeast cells in the exponential growth phase were maintained in the chemically defined solid medium McVeigh Morton (MMcM) [35], for seven days at 36°C and inoculated in liquid MMcM. A stock solution (1 mg/ml) containing sterile itraconazole (Sigma-Aldrich, St. Louis, MO, USA) was prepared in dimethylsulfoxide (DMSO). The final concentration of the solvent in the medium never exceeded 2% (v/v) and had no effect on the cell growth. From this stock solution, the drug was serially diluted in sterile MMcM (pH 7.0), producing a final concentration of 1.25-320 μg/ml (5–1260 mM). The drug concentration range was selected based on previous studies [36]. The controls without antifungal and DMSO were included. The concentrations of inoculums were determined by spectrophotometer using a yeast cell suspension in sterile 0.85% NaCl with 10% transmittance at 520 nm. The mixture was stirred to disperse aggregated cells. Yeast cells were collected from the liquid MMcM and counted in a Neubauer chamber. An initial inoculum containing 5 × 10^6^ cells/ml was collected, and 0.1 ml aliquots were added to 2.4 ml of MMcM containing the drug dilutions. The fungus was grown at 36°C under agitation at 150 rpm for five days. The IC_50_ was determined using measurements of the turbidity of the medium [37]. The experiments were processed in triplicate.

For viability experiments, yeast cells were grown in the presence or absence of 4 μg/ml (IC_50_) of itraconazole and were kept in liquid MMcM [35] for 1, 2, 3, 4 and 5 h at 36°C before the viability of the cells was determined by Trypan Blue method [38]. In brief, cells from all incubation times were incubated with a dye solution (0.1% Trypan Blue Stain) for 5 min at room temperature, and viability was assessed by counting viable and unviable cells in a Neubauer chamber.

### RDA: RNA extraction and cDNAs synthesis

*Paracoccidioides Pb*01 yeast cells were cultured in MMcM broth medium in the presence or absence of 4 μg/ml of itraconazole for 1 h and 2 h, corresponding to a viability of 95% and 85%, respectively. For RNA isolation, cells were harvested by centrifugation, washed in cold water and the RNA from driver and tester cultures were extracted with Trizol (Invitrogen, Carlsbad, CA, USA) according to the manufacturer’s instructions. RNA quality was assessed using the A_260nm_/A_280nm_ ratio. The RNA was treated with DNAse I RNAse-free (Invitrogen) to remove chromosomal DNA. The concentration and purity of RNA were determined by spectrophotometer, and RNA integrity was visualized after electrophoresis on 1.2% agarose gel. The RNAs were used to construct subtracted libraries and qRT-PCR experiments.

The cDNA fragments used for processing the RDA were generated according to the protocol previously described by Hubank and Schatz [39] and modified by Pastorian et al. [40]. Briefly, first-strand cDNA synthesis was performed with 1 μg total RNA, obtained from driver and tester cultures, using SuperScript III reverse transcriptase (Invitrogen). The first-strand cDNA obtained (3 μl) was used as template to synthesize the second-strand of cDNA. The cDNA was prepared using the SMART PCR cDNA synthesis kit (Clonetech Laboratories, Palo Alto, CA, USA).

### RDA: Subtractive hybridization

The cDNAs were digested with the restriction enzyme *Sau3*AI. Two successive rounds of subtraction employing different adapters (J-Bam and N-Bam, Table 2) were performed to enrich the differentially expressed sequences. Four cDNA-subtracted libraries were constructed. The cDNA libraries containing up-regulated genes were constructed from driver cDNA obtained from *Paracoccidioides Pb*01 yeast cells grown for 1 h and 2 h in MMcM medium and from tester cDNA, which was synthesized from RNA extracted from *Paracoccidioides Pb*01 yeast cells grown for 1 h and 2 h in MMcM medium plus itraconazole. The cDNA libraries containing down-regulated genes were constructed from driver cDNA, obtained from *Paracoccidioides Pb*01 yeast cells, grown for 1 h and 2 h in MMcM medium plus itraconazole and tester cDNA, which was synthesized from RNA extracted from *Paracoccidioides Pb*01 yeast cells grown for 1 h and 2 h in MMcM medium. The resulting products were purified using a GFX kit (GE Healthcare, Uppsala, Sweden). The tester-digested cDNA, from 1 h and 2 h samples, was linked to adapters (a 24-mer annealed to a 12-mer) and amplified by PCR.

**Table 2:** Oligonucleotide primers used in RDA assays and qRT-PCR

**Sequence name**	**Forward primer (5′-3′)**	**Reverse primer (5′-3′)**	**Amplicon size (bp)**
Glutathione S-transferase (*GST*)	GAACCGCAAACCCTAACCCT	ACAGCGGCTGAAAAGTCCCA	157
Chitin synthase regulator 2 (*CHSr*)	AGAGCTGCAGAATTAGGCCTT	TTTCGCCCGTTCATCTCCGT	140
Betaine aldehyde dehydrogenase (*BADH*)	GTTGAAGAGCCATTTGGTCC	CAGATCATTGGACCACACAGA	120
Cysteine desulfurase (*CYSD*)	CAACAGAAGAGATGGAGTATGA	AGCGAATGACACGTTGACACA	143
Ribulose-phosphate 3-epimerase (*RP3E*)	CAATGGATCGACCTGATATGG	GACCTCCGTCAACTTCGATG	141
Carnitine/acyl-carnitine carrier (*CAR*)	GAAGGCATTGCCAGGGGGT	CATTATGAACGGGGACGGTG	139
Glutamine amidotransferase subunit pdxT (*GLAT*)	TGAGAGACTTTGTCAAGAACCA	TGCGCGGATAAATACACCCAT	143
Mfs transporter (*MFS*)	CTAATTATGTTCTTTTGGGGTAC	GCATCGCCTATACCAACAAGA	136
C6 transcription factor (*CTF1B*)	CAAACCACTCGTCAACACAATC	GATTGCCTTGAGTCTGATAGAG	138
Acyl-CoA dehydrogenase (*ACAD*)	GAGAACGAGACGCCCGAAG	GTTGTAGTAAGGACTCTTGTAG	108
*GPR1/FUN34/YAAH* family protein	ACTGGCTGGGATGTGGGAG	TTCTTCTCCGTCATTTCCTTGA	141
Pyruvate kinase (*PK*)	ATGCGATGATAAATATCTCTACG	GACACTTGGCGCGGAGAGA	143
Diacylglycerol *o*-acyltransferase (*DGAT*)	TATTAGATATACCAAGTGGCCG	TACCCTGGGTTTGTATTCAATG	143
Isovaleryl-CoA dehydrogenase (*IVD*)	GATGTGGATTACCAACGGGC	TCATGCCAAGCTTGTCGAGTT	152
Ubiquitin-protein ligase (*UBI*)	GGAGGCATGCAGATCTTCGT	ACGACCGTCCTCAAGCTGC	168
Family integral membrane protein (*IMP*)	CGCCAGCAATCTGATTATCTC	AACCCAGCTGACCTTCATTAC	142
Heat shock protein (*HSP10*)	TCTTCCTCCCAGAGAGCGC	CAGGGCTGCCTCCATACTG	143
Heat shock protein (*HSP30*)	GGCCTTGACAGCATTCTGG	CTGGCGATAAAGGGCAGAAG	130
Heat shock protein (*HSP70*)	GCAGAAGGAGCTTGAAAGTGT	GTCAACCTCCTCGACAGTAG	181
ATP synthase f0 subunit 9 (*ATPS9*)	AAGCAGCGAAAATAATGGGATC	GCAAATAATCCTGTAGCTTCTG	181
Lanosterol 14 α-demethylase (*ERG11*)	CTGAGCTGTAGGGAAAAGTAC	TCCTCAGCGCAAACGTCCTT	131
C5,6-desaturase (*ERG3*)	GGAGAATATGTATACCAGCCC	ATCCAAGTGATGAGATACAGAG	128
Delta-24-sterol C-methyltransferase (*ERG6*)	GCTACTCTTACCCGACATTAC	AATGGGCAAGGTAATGTTCATG	142
C-22 sterol desaturase (*ERG5*)	GGTCCCATGTTCAAAATCCCT	AAATTTGTGGAAAACCGAGACG	123
C-4 methyl sterol oxidase (*ERG25*)	GGACCATGGCCTACCAAATC	GCGGAGTATTGGTGGTGGAT	129
cDNA*	AGCAGTGGTATCAACGACAGAGTACGCGGG		-
CDS*	AAGCAGTGGTATCAACGCAGAGTACT(30)N1N		-
PCRII*	AAGCAGTGGTATCAACGCAGAGT		-
JBam12*	GATCCGTTCATG		-
JBam24*	ACCGACGTCGACTATCCATGAACG		-
NBam12*	GATCCTCCCTCG		-
NBam24*	AGGCAACTGTGCTATCCGAGGGAG		-
RBam12*	GATCCTCGGTGA		-
RBam24*	AGCACTCTCCAGCCTCTCACCGAG		-
T7*	GTAATACGACTCACTATAGGGC		-
Oligo (dT)_15_*	AAGCAGTGGTATCAACGCAGAGTACT(30)N1N		-

*Primers used in RDA experiments.

For the generation of the differentially up- and down-regulated products, the tester and driver cDNAs of both conditions were mixed separately; the hybridization occurred at 67°C for 18 h and the amplification occurred by PCR using the oligonucleotide matching the 24-mer adaptor [41]. The successive rounds of subtraction and amplification were performed using hybridization tester-driver ratios of 1:10 and 1:100. Adapters (Table 2) were changed between cross-hybridization, and the different products were purified using the GFX kit. After the second subtractive reaction, the cDNA was purified and cloned directly into the pGEM-T Easy vector (Promega, Madison, WI, USA). *Escherichia coli* XL1 Blue competent cells were transformed with the ligation products. The plasmid DNAs were prepared from selected clones of subtracted libraries and sequenced with the ET Dye Terminator kit Dyenamic (GE Healthcare) in a MegaBACE 1000 DNA sequencer (GE Healthcare) using primers corresponding to the pGEM-T Easy vector.

### Processing and annotation of ESTs

The sequences of at least 75 nucleotides, with a PHRED score ≥ 20 were considered for the assembly and formation of clusters. The assembly of these ESTs was performed using CAP3 [42] and clustered to generate contigs and singlets, which were analyzed. All these tools were integrated in a specific pipeline (http://www.lbm.icb.ufg.br/pipelineUFG/). The annotation of genes was performed using the program Blast2GO (http://www.blast2go.org/), which provides a comparison between clusters of sequences obtained from public databases. The BLAST program from the National Center for Biotechnology Information (NCBI) (http://www.ncbi.lm.nih.gov/BLAST), processed with the non-redundant sequences (nr) GenBank and the nucleotide database generated from *Paracoccidioides* spp. structural genome (http://www.broad.mit.edu/annotation/genome/paracoccidioides_brasiliensis/MultiHome.html), was used for the annotation. The database sequence matches were considered significant at e-values ≤10^-5^. The program INTERPROSCAN (http://www.ebi.ac.uk/interpro/) [43] was used to obtain information about the domains present in clusters and the classification of families. The metabolic pathways were analyzed using maps obtained from the KEGG database (Kyoto Encyclopedia of Genes and Genomes) (http://www.genome.ad.jp/kegg) [44] with annotated EC numbers, and this information was used to help elucidate the function of ESTs. The Munich Information Center for Protein Sequences (MIPS) (http://mips.gsf.de/) was used to designate the functional categories. Additionally, sequences were grouped into functional categories using the PEDANT 3 database (http://pedant.helmholtz-muenchen.de/index.jsp).

### Analysis of RNA transcripts by qRT-PCR

An aliquot of RNA from treated and untreated samples was used to perform reverse transcription qRT-PCR. Total RNAs from *Paracoccidioides Pb*01 yeast cells cultured in the presence or absence of itraconazole were obtained as previously described, in independent experiments from those used in the RDA assays. After treatment with DNAse, cDNAs were synthesized from total RNA using Superscript III reverse transcriptase (Invitrogen) and oligo (dT)_15_ primer according to the supplier’s instructions. Gene-specific primers were designed for the selected genes and for the control gene, α-tubulin, using Primer Express software (Applied Biosystems, Foster City, CA, USA) (Table 2). qRT-PCRs were performed in triplicate in a StepOnePlus™ real time PCR system (Applied Biosystems). The PCR thermal cycling program consisted of 40 cycles of 95°C for 15 sec; 60°C for 1 min. The SYBR green PCR master mix (Applied Biosystems) was supplemented with 1 ρmol of each gene-specific oligonucleotide and 40 ng of template cDNA in a final volume of 20 μl. A curve melting analysis was performed to confirm the amplification of a single PCR product. The data were normalized to the α-tubulin transcript amplified in each set of qRT-PCR experiments. A no-template control was included. Samples of each cDNA were pooled and serially diluted 1:5 to generate a relative standard curve. Relative expression levels of genes were calculated using the standard curve method for relative quantification [45]. Statistical comparisons were performed using Student’s *t*-test and samples with p-values < 0.05 were considered statistically significant. The specific sense and antisense primers are listed in Table 2. Itraconazole-regulated transcripts were selected for qRT-PCR validation assays.

### Preparation of protein extracts and validation of data obtained by specific activity of Glutathione S-Transferase (GST)

GST activity was measured with GST assay kit (Sigma-Aldrich). Briefly, the GST Assay Kit employs 1-Chloro-2,4-dinitrobenzene (CDNB) to produce 1-glutathionyl-2,4-dinitrobenzene (GS-DNB) by conjugation of the thiol group of glutathione (GSH). The reaction product GS-DNB absorbs at 340 nm, and the rate of increase in the absorption is directly proportional to the GST activity of the sample.

Protein extracts from *Paracoccidioides Pb*18 yeast cells were prepared by inoculating 50 ml of Fava Netto’s liquid medium with 10^6^ cells/ml. Cultures were incubated overnight at 36°C with gentle shaking for 16 h. Cells were centrifuged at 5,000 x *g* for 5 min and transferred into MMcM media containing itraconazole for 1 h. Control cells were incubated in MMcM without drug. The cells were centrifuged at 10,000 x *g* for 15 min at 4°C, frozen in liquid nitrogen and disrupted by maceration [46]. Extraction buffer (20 mM Tris–HCl pH 8.8; 2 mM CaCl_2_) containing a mixture of protease inhibitors (serine, cysteine and calpain inhibitors) (GE Healthcare) was added to the yeast cells. After the addition of glass beads (0.45 mm), the cells were lysed in a bead-beater, followed by centrifugation at 10,000 x *g* for 15 min at 4°C. The supernatant was collected, and the protein concentrations were determined using Bradford reagent (Sigma-Aldrich). The samples were stored in aliquots at -80°C.

The increase in absorbance is directly proportional to the GST activity. The GST-specific activity is defined as mmol of GS-DNB per mg of total protein per min (mmol/mg/min). The enzymatic activity results represent the mean of three independent determinations, and statistical comparisons were performed using Student’s *t* test. The samples with *p*-values ≤0.05 were considered statistically significant.

### Sterol quantification method

The quantification of total intracellular ergosterol was performed as previously described [47], with slight modifications. Cell extracts from *Paracoccidioides Pb*01 and *Pb*18 yeast cells were prepared as already described above. Five ml of 25% alcoholic potassium hydroxide solution (25 g KOH and 35 ml sterile distilled water added to 100 ml 100% ethanol) was added to each tube, and the samples were mixed on a vortex for 2 min. The cell suspensions were incubated in an 85°C water bath for 3 h and allowed to cool to room temperature. Sterols were extracted by addition of 2 ml of sterile distilled water and 5 ml *n*-heptane (Sigma-Aldrich), followed by vigorous mixing in a vortex mixer for 5 min. The samples were kept at room temperature for 1 to 2 h to allow the phases to separate or were stored at 4°C overnight. One ml of the heptane layer (containing ergosterol) was transferred to a 1.5 ml quartz cuvette and analyzed spectrophotometrically by scanning at wavelengths between 200 and 300 nm. If necessary, the samples were diluted five fold with 100% ethanol and reanalyzed. The ergosterol content as a percentage of the wet cell weight was calculated by the following equations: value 1 = [(A_281.5_/290) × *F*]/wet cell weight, value 2 = [(A_230_/518) × *F*]/wet cell weight, and percent ergosterol = value 1 - value 2. *F* is the factor for dilution in ethanol, and 290 and 518 are fixed values determined for crystalline ergosterol and 24(28) dihydroergosterol, respectively.

### Fluorescence microscopy

Filipin stained samples were prepared using a previously described protocol for fixing and staining filamentous fungi [48]. *Paracoccidioides Pb*01 and *Pb*18 yeast cells were prepared by inoculating 50 ml of Fava Netto’s liquid medium with 10^8^ cells/ml. Cultures were incubated overnight at 36°C under gentle shaking for 16 h. Cells were centrifuged at 5,000 × *g* for 5 min and transferred in MMcM media containing itraconazole. Control cells were incubated in MMcM without drug. The fungus was then removed and fixed for 30 min in 3.7% formaldehyde and rinsed with ddH_2_O. A 5 mg/ml stock solution of filipin (Sigma-Aldrich) dissolved in DMSO was diluted to 25 μg/ml and used to stain the fixed samples for 10 min. Samples were then rinsed with ddH_2_O, mounted on a microscope slide and sealed with nail varnish.

### BALB/c mice infection with *Paracoccidioides Pb*18

The animals were bred at the Universidade Federal de Goiás animal facility under specific-pathogen-free conditions. All animal experiments were performed in accordance with the international rules for animal experimentation. The animal protocol was approved by the Universidade Federal de Goiás committee of the ethical treatment of animals (Number: 008/11 CEUA-UFG).

Female BALB/c mice, 8–12 weeks old, were inoculated with 1 × 10^7^ of *Paracoccidioides Pb*18 yeast cells growth in liquid MMcM. In brief, yeast cell suspension in the 7th day of *in vitro* growth were washed in PBS 1× and inoculated intraperitoneally in mice. The mice were divided into three groups of five animals to be sacrificed 21 days post-challenge. Each group was subdivided by treatment options as follows: five uninfected mice (negative controls), five yeast cell-infected mice (positive controls) and five yeast cell-infected mice treated with itraconazole starting at the third week post-infection. The animals were sacrificed in the sixth week after infection. The spleens were removed and homogenized in 5 ml of sterile PBS 1X. The homogenized sample was plated in brain heart infusion agar supplemented with 4% (v/v) fetal calf serum and 2% (w/v) glucose. The plates were incubated at 36°C and colony-forming units were determined after 20 days.

Gene expression analyses of *Paracoccidioides Pb*18 from infected mice were performed by isolating yeast cells from spleens as previously described with minor modifications [49]. The spleens of infected mice were homogenized in 1× PBS using a tissue grinder. The homogenate was then filtered using nylon mesh to remove large pieces of animal tissue. The sample was frozen in liquid nitrogen and then centrifuged at 500 × *g* for 5 min to remove any remaining animal tissue. Next, the sample was centrifuged at 7,000 × *g* for 15 min to isolate fungal cells. Total RNA was extracted from recovered cells using TRIZOL reagent (Invitrogen), according to the manufacturer’s instructions. RNA was used to perform qRT-PCR as described above.

## Competing interests

The authors declare that they have no competing interests.

## Authors’ contributions

BRSN performed RNA extraction and Construction of cDNA libraries subtractive, annotation of ESTs, Analysis of RNA transcripts by qRT-PCR, Analysis of ERG transcripts by qRT-PCR, Preparation of protein extracts and specific activity of Glutathione S-Transferase (GST), Analysis of the ergosterol level, Fluorescence microscopy, and model for the *Paracoccidioides* spp. adaptation to the itraconazole. PFZC perfomed experiment sub-inhibitory concentration. WSM perfomed processing of ESTs. BRSN and AMB made BALB/c mice infection with *Paracoccidioides Pb*18. BRSN, AMB, CMAS and MP contributed to the discussion of the data and preparation of the manuscript. MP conceived, designed and coordinated the study. All authors contributed to the discussion of results. All the authors have read and approved the final manuscript.
